# 3D imaging of cells in scaffolds: direct labelling for micro CT

**DOI:** 10.1007/s10856-018-6089-6

**Published:** 2018-06-12

**Authors:** David V. Shepherd, Jennifer H. Shepherd, Serena M. Best, Ruth E. Cameron

**Affiliations:** Department of Materials Science and Metallurgy, University of Cambridge, 27 Charles Babbage Road, Cambridge, CB3 0FS UK

## Abstract

The development of in-vitro techniques to characterise the behaviour of cells in biomedical scaffolds is a rapidly developing field. However, until now it has not been possible to visualise, directly in 3D, the extent of cell migration using a desktop X-ray microCT. This paper describes a new technique based on cell labelling with a radio opacifier (barium sulphate), which permits cell tracking without the need for destructive sample preparation. The ability to track cells is highlighted via a comparison of cell migration through demonstrator lyophilised collagen scaffolds with contrasting pore size and interconnectivity. The results demonstrate the ease with which the technique can be used to characterise the effects of scaffold architecture on cell infiltration.

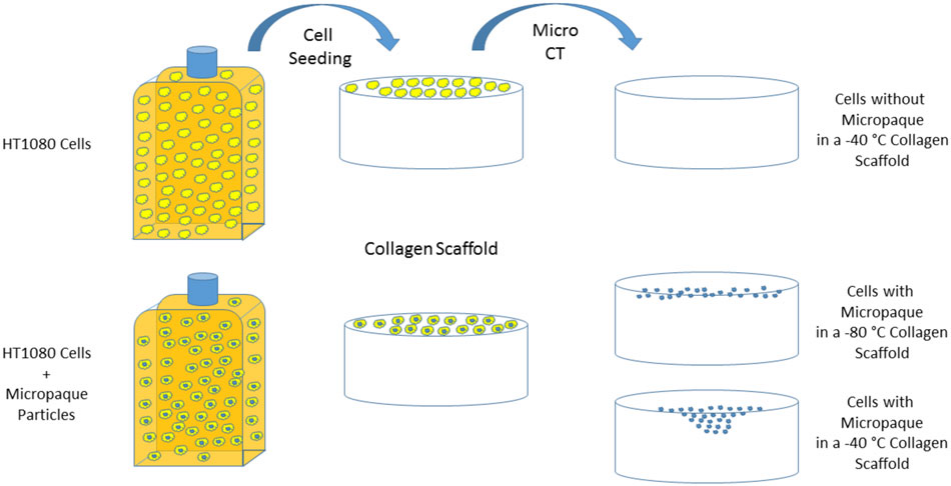

## Introduction

The design and characterisation of three-dimensional architectures for optimised cell delivery is an essential component of tissue engineering [1] and understanding the ability of cells to populate the scaffold in-vitro and in-vivo is of key importance [2]. However, laboratory processes for in-situ cell tracking are restricted to 2D sections and are usually disruptive and often destructive due to the need for sectioning and staining [3]. Therefore, there is an urgent need in the field of cell tracking, for a technique which minimises post-culture intervention and reduces cell damage. X-ray microcomputed tomography (microCT) has previously been explored as a method for three-dimensional analysis of cell-containing scaffolds, but has required the use of toxic fixatives (such as osmium tetroxide) [4], complex post-analysis (removing the substrate to reveal cells) [5] or the use of expensive synchrotron sources [6].

We have developed a technique that offers to revolutionise the way that cells are imaged in scaffolds. By adapting a technique used *in vivo*, we demonstrate that we are able to image, in 3D, the infiltration of cells within a scaffold using a desktop microCT, using a simple and relatively cheap method without the requirement to manipulate data once captured. This work demonstrates a novel route for cell tracking, with the potential for live cell imaging in scaffolds in the future.

## Method

### Scaffold production

Collagen scaffolds were produced through lyophilisation according to a method described previously [3]. Briefly 1% collagen suspensions were produced by blending 1 g of collagen (Bovine, Devro) in 100 ml of 0.05 M acetic acid. A 48 well plate was filled with 0.8 ml of the collagen slurry in each well and pre-frozen at −40 or −80 °C for 1 h before freeze-drying. After this, the scaffolds were cross-linked using EDC/NHS before freeze drying again.

### Cell culture

HT 1080 cells were pre-cultured for 72 h in the absence and also in the presence of Micropaque (Guerbet), a solution of barium sulphate used in medical CT scans, at 3.5 μl Micropaque per ml of cell culture media (DMEM and Fetal Calf Serum, L-Glutamine–Penicillin–Streptomycin solution) (Sigma-Aldrich) [7]. Cells were subsequently washed in PBS (Gibco) trypsinised (trypsin, Sigma-Aldrich) and then seeded onto the top surface of scaffolds at 80,000 cells per scaffold in a 15 μl bead. The cells were allowed to adhere to the scaffold for 2 h, before being transferred to a new well plate and the wells flooded with cell culture media only. Scaffolds were removed at days 1, 4 and 7 for imaging and at each time point, the media was changed for the remaining scaffolds. Control samples (without cells) were also produced.

At each time point, scaffolds were removed from the culture media and washed using PBS. They were then fixed using ultrapure glutaraldehyde (5% solution in DI Water) (Sigma, UK) for 30 min. Next, the scaffolds were washed in DI water three times, 5 min per wash before being placed onto a pre-frozen shelf (−40 °C) of a freeze drier and dried. After the cycle the samples were removed and washed three times, 5 min per wash, and placed back into the freeze drier. This removed any salts remaining in the scaffolds. The washing step was repeated a further two times.

### Micro CT

#### Scanning and reconstruction

All samples were scanned using Micro CT (Bruker Skyscan 1272). An operating voltage of 25 kV was used with a pixel size of 2.93 μm. A stepsize of 0.2° and frame averaging of 2. Reconstructions were carried out using NRecon (Bruker Micro CT) using a full cone beam Feldkamp reconstruction algorithm with greyscale limits defined automatically.

#### Analysis

The effect of the different shelf temperatures on scaffold architecture characteristics was investigated using the Bruker CTAn programme. For each freezing condition, nine 1 mm^3^ regions of interest (ROIs) were selected (3 regions of interest within 3 samples for each sample type). Thresholding of these ROIs was carried out using automatic Otsu thresholding followed by a sweep de-speckle process. Overall porosity was determined along with mean pore size using a three-dimensional sphere-fitting method. Scaffold interconnectivity is a key predictor for cell migration and measurements were made to identify inaccessible regions of a scaffold ROI according to a given definition of the minimum size of a pore interconnection; the accessible pore space is determined for a virtual object, with diameter equal to the minimum connection size. A three dimensional shrink-wrap analysis was carried out (CTAn, Bruker Micro-CT) for progressively larger diameter objects and, for each diameter, the % interconnectivity was defined by:

${\mathrm {Interconnectivity}}{\mathrm{ = }}\frac{{v - v_s}}{{v - v_m}},$where v = total VOI, v_s_ = inaccessible scaffold volume after shrink wrap, v_m_ = volume of solid material within VOI.

#### Imaging

To visualise the scaffolds, reconstructed images were volume-rendered within Bruker Micro-CT’s CT Vox programme. A representative image was selected and a function defined which altered the opacity profile such that, background noise was reduced to transparency. This then meant that the scaffold structure and cells clearly observed. This same transfer function was applied to each of the models. With this simple transfer function the Micropaque and thus the cells could be identified clearly, without the need for manipulation. A more complex transfer function was also considered whereby the grey-scale range of the micropaque material was highlighted through colour selection.

## Results

**Fig. 1:**
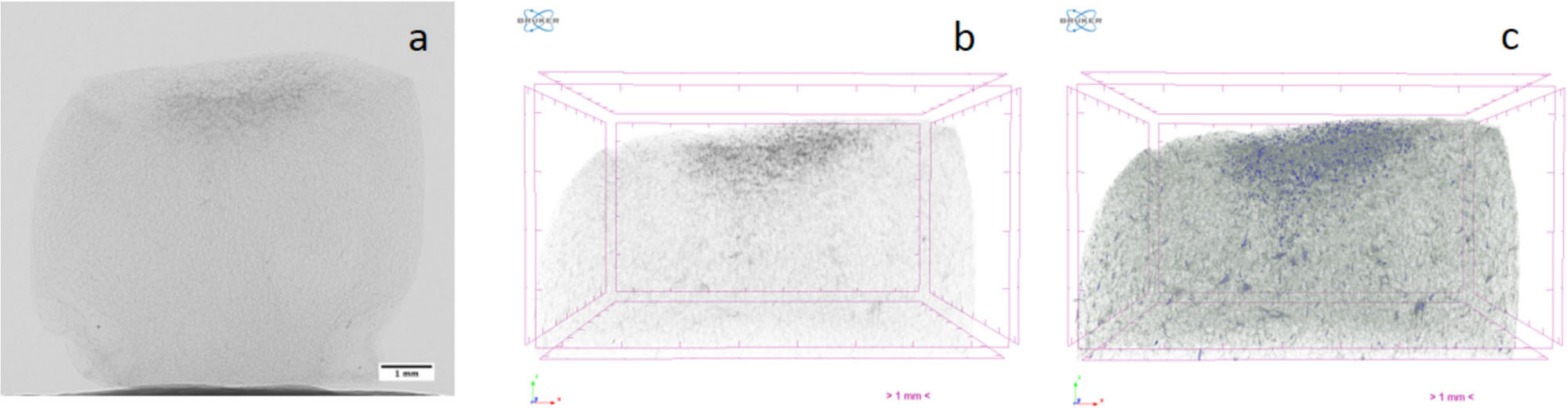
**a** Shadow Projection as visualised in NRecon; **b** reconstructed image with simple Transfer Function; **c** reconstructed image with Transfer Function to apply colour

The cells took up the Micropaque successfully and were clearly visible in the collagen scaffolds. Figure 1a shows a shadow projection of a collagen scaffold produced at −40 °C with cells pre-cultured in the presence of micropaque seeded onto the top surface and cultured for one day (as seen in NRecon before reconstruction). Figure 1b shows an image after application of the simple transfer function. Figure 1c demonstrates the effect of colour selection within the transfer function.

**Fig. 2:**
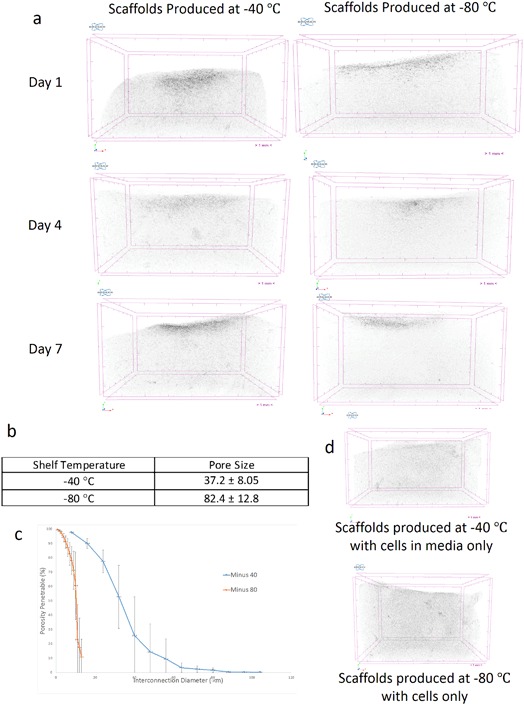
Micro CT Reconstructed images of the HT1080 cells cultured in the presence of Micropaque infiltrating into collagen scaffolds over 7 days (**a**). The scaffolds used had differing pore structures leading to different pore sizes (**b**) and varying interconnectivity (**c**). When no contrast agent was used with the cells the scaffolds appear identical to scaffolds where no cells are present (**d**)

Figure 2a is a composite image of scaffolds produced at −40 and −80 °C that have been seeded with and without cells that were pre-cultured with Micropaque viewed in CTVox after the transfer function has been applied. From the images it is clear that cells can only be seen where Micropaque is present. Only where cells were present, did the scaffold show areas of enhanced radio opacity (Fig. 2d).

The table included in Fig. 2b shows the effect of varying the shelf temperature on the pore sizes of the collagen scaffolds. The lower freezing temperature resulted in a significantly higher mean pore size. Figure 2c demonstrates that while the pore size for the −80 °C scaffold was larger, the interconnectivity of the scaffold produced at −40 °C was significantly higher than that of the scaffolds produced at −80 °C.

## Discussion

The presence of barium sulphate particles within the Micropaque was evident from microCT images (Fig. 1). The shadow projection (*1a*) shows the presence of the Micropaque in the cells situated close to the original seeding site on the scaffold. It is possible to observe the entire colony once all of the shadow projections have been combined and reconstructed using the transfer function (Fig. 1b) further enhancing the clarity of the image. The images also highlight density variations within the scaffolds and this effect is more pronounced with post-processing by applying a colour filter (Fig. 1c). An interesting observation is that there appear to be fewer cells present in the scaffolds by day 7 (since it can be noted that the contrast visible within the scaffold has reduced). There are two possible explanations for this. The first is that Micropaque concentration would reduce with each cell division and hence the cell X-Ray opacity would decrease over time. Alternatively, cells may become quiescent, or the opacifier uptake may have led to slow cell apoptosis. However, the effects of early-stage migration are clear.

Two lyophilisation conditions were chosen to create demonstrator scaffolds. By varying the temperature of the pre-frozen shelf, the pore sizes could be controlled (Fig. 2b) with a counter-influence of interconnectivity (Fig. 2c). It might be expected that where interconnectivity is high (−40 °C) the cells would be able to penetrate into the scaffold - and this was confirmed, as shown in Fig. 1a. For the structures produced at −80 °C, it was observed that the cells initially travelled across the scaffold before penetrating at a later time point, but by day 7, the imaging technique could be used to demonstrate that, at later time points, there was very little difference in the distance travelled by the cells in either type of scaffold.

## Conclusion

The work presented demonstrates clearly that cells can be observed, directly, within 3D environments *in vitro*. The method is reproducible and easy and involves no post-processing to locate the cells in scaffolds. The ability to track and map colonies within 3D scaffolds will enable design of constructs for optimised cell migration.

## Electronic supplementary material

Supplementary Information
